# Modelling Reveals Kinetic Advantages of Co-Transcriptional Splicing

**DOI:** 10.1371/journal.pcbi.1002215

**Published:** 2011-10-13

**Authors:** Stuart Aitken, Ross D. Alexander, Jean D. Beggs

**Affiliations:** 1 Centre for Systems Biology, University of Edinburgh, Edinburgh, United Kingdom; 2 Wellcome Trust Centre for Cell Biology, University of Edinburgh, Edinburgh, United Kingdom; Center for Genomic Regulation, Spain

## Abstract

Messenger RNA splicing is an essential and complex process for the removal of intron sequences. Whereas the composition of the splicing machinery is mostly known, the kinetics of splicing, the catalytic activity of splicing factors and the interdependency of transcription, splicing and mRNA 3′ end formation are less well understood. We propose a stochastic model of splicing kinetics that explains data obtained from high-resolution kinetic analyses of transcription, splicing and 3′ end formation during induction of an intron-containing reporter gene in budding yeast. Modelling reveals co-transcriptional splicing to be the most probable and most efficient splicing pathway for the reporter transcripts, due in part to a positive feedback mechanism for co-transcriptional second step splicing. Model comparison is used to assess the alternative representations of reactions. Modelling also indicates the functional coupling of transcription and splicing, because both the rate of initiation of transcription and the probability that step one of splicing occurs co-transcriptionally are reduced, when the second step of splicing is abolished in a mutant reporter.

## Author Summary

The coding information for the synthesis of proteins in mammalian cells is first transcribed from DNA to messenger RNA (mRNA), before being translated from mRNA to protein. Each step is complex, and subject to regulation. Certain sequences of DNA must be skipped in order to generate a functional protein, and these sequences, known as introns, are removed from the mRNA by the process of splicing. Splicing is well understood in terms of the proteins and complexes that are involved, but the rates of reactions, and models for the splicing pathways, have not yet been established. We present a model of splicing in yeast that accounts for the possibilities that splicing may take place while the mRNA is in the process of being created, as well as the possibility that splicing takes place once mRNA transcription is complete. We assign rates to the reactions in the pathway, and show that co-transcriptional splicing is the preferred pathway. In order to reach these conclusions, we compare a number of alternative models by a quantitative computational method. Our analysis relies on the quantitative measurement of messenger RNA in live cells - this is a major challenge in itself that has only recently been addressed.

## Introduction

The splicing of precursor messenger RNA (pre-mRNA) is an essential process in the expression of most eukaryotic genes. The five small nuclear ribonucleoproteins (snRNPs) and the many non-snRNP-associated proteins that constitute the splicing machinery, assemble anew on each precursor RNA to form the spliceosome complex that catalyses the two chemical reactions of splicing [1]. Both the spliceosome components and the spliceosome assembly process are largely conserved between human and yeast. The complexity of the spliceosome is indicated by the 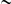
170 proteins that are associated with it [1]. Adding to the complexity, splicing may occur partly, or entirely, concurrently with transcription. In eukaryotes, the interaction of the spliceosome with the precursor RNA can be considered to be an allosteric cascade in which early recognition steps induce conformational changes required for subsequent steps and for catalytic activation (reviewed by [2]). However, the wealth of knowledge of molecular interactions, obtained mainly through extensive biochemical and genetic analyses, has yet to be formalised as a systems model of transcription and splicing.

**Figure 1:**
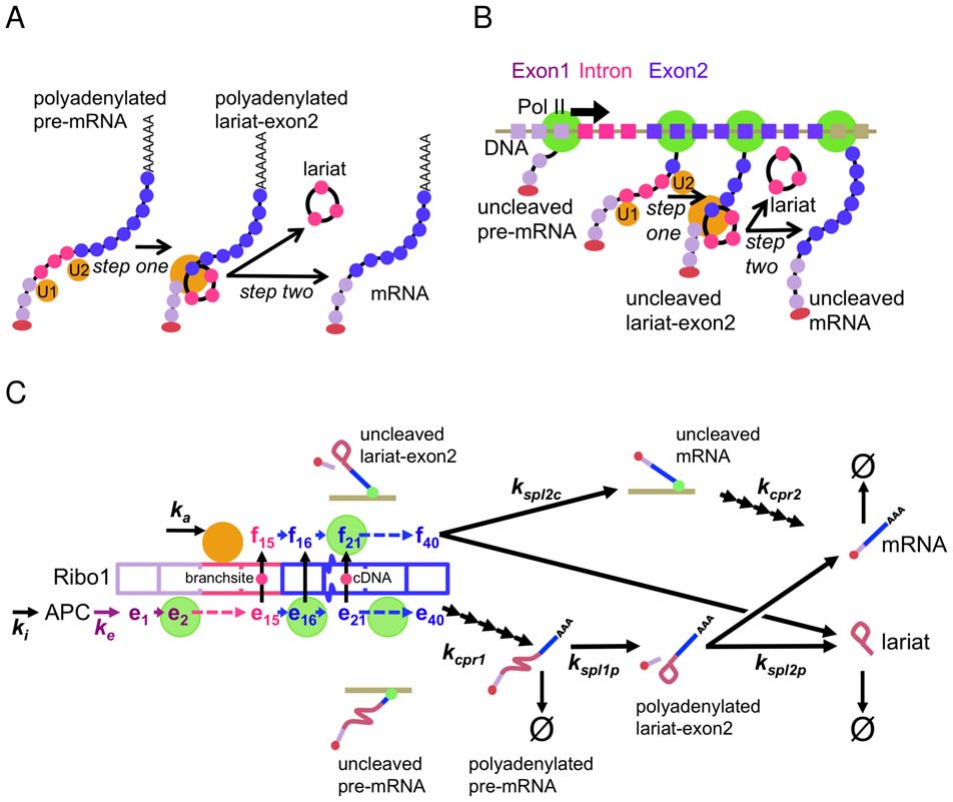
Post-transcriptional and co-transcriptional splicing. A) Spliceosome assembly begins with the U1 and U2 snRNPs. On the post-transcriptional splicing pathway, polyadenylated pre-mRNA is spliced (step one) to produce polyadenylated lariat-exon2, from which mature mRNA results on completion of step two. B) Splicing can occur during transcript elongation on the co-transcriptional splicing pathway. The RNA polymerase Pol II (shown in green) transcribes Exon1, the Intron and Exon2 (colours of the RNA indicate the corresponding region of the DNA, and the red oval indicates the cap on the 5′ end of the RNA). Splicing can occur after the transcription of specific sequences that trigger the assembly and activation of the spliceosome on the RNA. C) The transcription and splicing pathway. A polymerase proceeds from the active promoter complex (APC) through 15 irreversible steps that represent the transcription of nucleotides in Exon 1 and the Intron up until the branchsite. From the branchsite (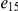) until the end of Exon 2, a transition can be made to the co-transcriptional splicing path 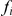 as the first step of splicing can occur. The characteristics of post-transcriptional splicing steps one and two (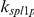 and 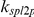), and co-transcriptional splicing step two (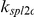) are determined by model comparison.

Spliceosome assembly is thought to occur via a series of events with many points of regulation [3]. In the first step, U1 snRNP binds to the 5′ splice site (5′SS), followed by the U2 snRNP at the branchsite. The U4, U5 and U6 snRNPs join as a tri-snRNP complex and, after the association of other, non-snRNP proteins, the spliceosome complex is activated for the first chemical step of splicing. The 5′ splice site is cleaved and, simultaneously, the 5′ end of the intron becomes covalently attached to the branchsite to form a branched, lariat structure. In the second step, the 3′ splice site (3′SS) is cleaved, which excises the intron, and the exons are joined to produce the mature mRNA. Between the two steps of splicing, a conformational change is required in the catalytic centre of the spliceosome [4], and at several stages during the cycle of spliceosome assembly, splicing and spliceosome dissociation, proofreading mechanisms are thought to operate [5]. Nascent transcripts also have to be matured at their 3′ end, by cleavage and polyadenylation. Figure 1 A illustrates spliceosome assembly and the two steps of splicing for a pre-mRNA with one intron that has already been polyadenylated and released from the DNA template.

Splicing can also occur co-transcriptionally, prior to 3′ end maturation (Figure 1 B), and there is considerable experimental evidence for functional coupling of transcription, splicing and 3′ end maturation *in vivo* [6]–[12]. However, little is known about the impact of coupling on kinetic rates. Splicing has been modelled, but not to the same level of detail as transcription, and models of transcription have yet to fully incorporate the splicing reaction. Quantifying the dynamics of these processes remains a challenge [13], and modelling may have an important role to play in distinguishing functional dependencies from coincidental and contemporaneous effects, and in identifying and characterising the interactions that effect coupling.

Existing models of splicing have allowed splicing efficiency to be defined [14], and have shown that transcription by RNA polymerase II (Pol II) greatly increases splicing efficiency in comparison with transcription by T7 polymerase [15]. A correlation between splicing efficiency and the pausing of Pol II on short terminal exons has been reported [11]. Splicing has been represented as a single irreversible reaction that creates the product mRNA from pre-mRNA [11], [14], and as a single irreversible reaction that creates mRNA from the pre-mRNA+spliceosome complex [15]. To-date, steps one and two of splicing have not been modelled as separate reactions, nor have the co- and post-transcriptional splicing pathways been distinguished. Further insights into splicing can be expected by more detailed modelling and analysis.

As noted above, splicing can occur during messenger RNA transcription. Transcription begins with the assembly of the pre-initiation complex at the promoter. This complex includes Pol II, which, after initiation, begins the transcript elongation process that transcribes DNA into RNA. Early in elongation, the pre-mRNA is capped at its 5′ end by the capping enzymes. Elongation involves a sequence of many hundreds of individual polymerisation reactions, and hence the time required to complete the elongation of a transcript is predicted to have less variability than a single-step process with an equivalent rate [16], [17]. The mature 3′ end of the RNA is formed by an endonucleolytic cleavage at the so-called poly A site and the newly formed 3′ end is extended by polyadenylation (reviewed by [18]). The elongation process and the 3′ end formation steps can also be accounted for when modelling transcription [16].

The recruitment of Pol II enzymes and spliceosomal proteins are important steps in transcription and splicing, but are not believed to be rate limiting under normal conditions. Kinetic studies of Pol II complexes indicate that a minority of them are actively involved in transcription at any given time. The remainder move by diffusion through the nucleus [19], as do the product mRNA molecules [20]. Three kinetically distinct populations of Pol II have been identified at the site of transcription; those bound to the promoter, those initiating transcription, and those engaged in elongation [21]. The movement of the spliceosomal proteins that catalyse the splicing reactions can be modelled as Brownian diffusion [22]: these RNPs move continuously throughout the nucleus independently of transcription and splicing.

We have developed a stochastic model that represents splicing in the context of transcript elongation and RNA 3′ end maturation, as shown diagrammatically in Figure 1 C. (All pathway models are provided as files in Dataset S1.) A stochastic formulation allows the effects of small numbers of molecules to be explored, and simulations of the model can be averaged in order to obtain the population mean over time. Experimental values for the model species (population averages in copies/cell), including fully-spliced mRNA (see Materials and Methods) and two precursor species in both 3′ uncleaved and cleaved/polyadenylated forms, have been obtained by a rapid sampling protocol that is capable of capturing transient species [23]. We first describe the structure of the pathway, then present the data, and subsequently discuss alternative representations of the steps in the RNA pathway in the light of the data. The simplest description that might be adopted for the elongation, 3′ end formation and splicing steps is a single irreversible reaction. However, we find this provides a poor fit to the available data, and consequently a number of alternative representations for these reactions are considered. The extent to which the alternative pathways fit the data is assessed by the Akaike information criterion (AIC) for optimal parameter choices.

## Results

### Modelling transcription and splicing

We propose a multistep model for transcription by dividing the gene into sections to be transcribed. Each section (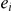) of the reporter DNA represents approximately 30 nucleotides, corresponding to the footprint of Pol II on the DNA [24]. As the length of the Ribo1 reporter (described below) used in the experimental studies is 1240 bases, we define 40 sections of DNA: 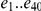. Each section of DNA can be occupied by at most one Pol II, and the progression of Pol II from the 5′ to the 3′ end of the gene is equated with successful extension of the transcript. The number of sections of DNA defines an upper limit on Pol II occupancy, and can limit the effective rate at which a Pol II can complete elongation. Beginning with the initiation of transcription, the 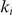 reaction (see Figure 1 C) places a Pol II enzyme in the active promoter complex (APC) when the gene is active. Thereafter, this Pol II can progress along the gene at elongation rate 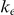 (the number of sections of DNA transcribed per unit of time). Letting the rate of polymerisation of nucleotides be 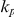 (the number of nucleotides incorporated per unit of time): 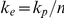. (Equivalently, the mean time for *n* polymerisation events: 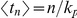, equals the mean time for one elongation event: 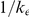). This multistep model of elongation is comparable with the kinetic model of Pol I elongation proposed in [25]. The pathway proposed here does not include a transition between active and inactive states of the promoter, as the rapid rate of mRNA production does not indicate that the promoter switches off during the period immediately after induction. However, such a transition is needed to explain the mRNA distribution in steady state [26] and can easily be included in this model.

Kinetic competition between splicing and elongation has been discussed extensively [8], [27], [28], and is modelled here as taking place at the sections of DNA after the branchsite. In these sections, the occurrence of the first step of splicing of an RNA is represented in the model by a change of state of the associated Pol II, which can make a transition to the co-transcriptional splicing path 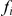. Sections 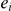 and 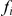 represent the same *n* nucleotides of the DNA and so at most one of these sections can be occupied (by at most one Pol II). The rate for the transition between splicing pathways is 0 prior to the completion of the splicing activation process. The splicing activation process is triggered at rate 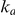 when the gene switches on. When splicing is active, the transition rate is 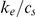, where 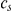 is a constant that determines the ratio of the competing reactions (elongation and splicing) and thereby the probability of co-transcriptional splicing. Activation of co-transcriptional splicing involves co-transcriptional spliceosome assembly as well as the first step splicing reaction (i.e. co-transcriptional spliceosome assembly alone is not sufficient). Each Pol II completes elongation either at 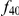, having completed the first step of splicing, or at 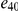 having failed to do so. Subsequently, on the post-transcriptional path, 3′ end maturation (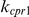) produces polyadenylated pre-mRNA, step one of splicing (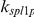) produces polyadenylated lariat-exon2, and step two of splicing (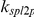) produces mature mRNA and lariat, as indicated in Figure 1 C. On the co-transcriptional pathway, the second step of splicing (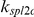) produces uncleaved mRNA and lariat, and 3′ end maturation (3′ cleavage, polyadenylation and release; 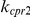), produces mature mRNA. It is important to note that the species measured experimentally are pre-mRNA, lariat-exon2 (the branched lariat structure) and mRNA, and that the uncleaved and polyadenylated forms can also be distinguished (as illustrated in in Figure 1 C). The assays for these species are described below.

Initial estimates for some parameters can be obtained from the literature: the rate of initiation of transcription in yeast has been estimated as 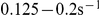 [29], [30]. Polymerisation rates in the mammalian nucleus of up to 72 nucleotides/s have been reported for polymerases that do not pause. This is a significant increase on earlier estimates of 18–42 nucleotides/s [13] that may reflect an average or effective rate. A Pol I elongation rate of 90 nucleotides/s has been reported [25]. The time for pre-mRNA 3′ cleavage in HIV-1 has been reported to be 55 s, with release taking 9 s [16]. The probability of co-transcriptional splicing is not known, and this, along with precise values for all other parameters, will be inferred from fitting the pathway to the data.

### Splicing and 3′ end cleavage assays

**Figure 2:**
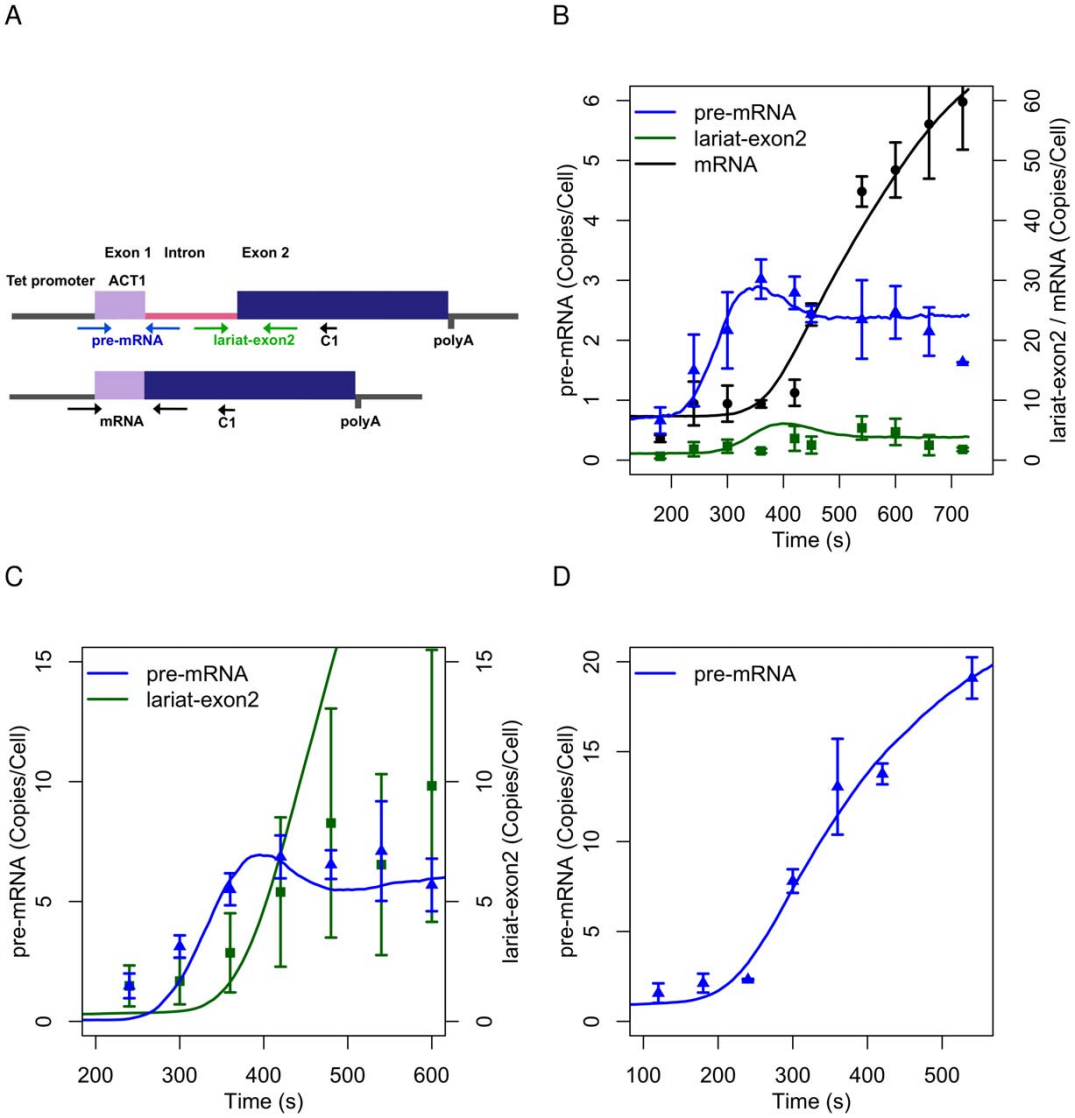
The Ribo1 reporter and its response to doxycycline. (A) Schematic of the promoter, intron and exons of Ribo1. The positions of the RT-qPCR primers are shown by the arrow C1. (B) Induction and splicing of Ribo1 measured by RT-qPCR. (C) Induction and step 1 splicing of 3′SSRibo1. Step two of splicing is blocked and therefore mRNA is not produced. (D) Induction of 5′SSRibo1. Step one of splicing is blocked and therefore neither lariat-exon2 nor mRNA is produced. Points indicate pre-mRNA (blue), lariat-exon2 (green) and mRNA (black) data. Error bars show the standard error of three biological replicates. Solid lines are model predictions.

The pathway was developed to explain data from the Ribo1 reporter [23]. Ribo1 is a chimeric yeast gene that contains the single intron from the *ACT1* gene and the 3′ end processing signal from *PGK1*, as shown in Figure 2 A (modified from [31]). The reporter gene is integrated in the genome, transcribed under the control of a doxycycline-responsive promoter in a doxycycline-inducible strain of *Saccharomyces cerevisiae*. By modelling splicing in this reporter, we aim to define the splicing pathways and to quantify reaction rates. The impact of splice site mutations on the coupling between splicing and transcription can also be explored.

Three replicate experiments were performed in which doxycycline was added to a culture to induce reporter gene expression, and transcript levels were measured by reverse transcription and real-time quantitative PCR (RT-qPCR; see Materials and Methods). A time series of values was obtained for accumulation of pre-mRNA, lariat-exon2, and mRNA. The RT-qPCR data were converted to copies per cell (see Materials and Methods; [23]), which allows a quantitative comparison of data obtained for the different RNA species and from different cultures. The merged time series derived from three biological replicates for Ribo1 is shown in Figure 2 B (referred to as Expt 1). In the 120 s interval 420 s–540 s after the addition of doxycycline to the cell culture, the level of Ribo1 mRNA increases from 11 to 45 copies/cell (Figure 2 B). Messenger RNA then reaches 60 copies/cell, on average, 180 s later. The high level of mRNA is notable, as is the rapid rate of transcript synthesis. The delay between the rise in pre-mRNA and the rise in mRNA may indicate a slow, or delayed, splicing reaction. In the substantive phase of transcriptional activity (after 420 s in Figure 2 B), the levels of pre-mRNA and lariat intermediate are only a fraction of the mature mRNA species which shows that the first and second steps of splicing must be rapid.

To investigate the effects of blocking the first or second step of splicing, two modified Ribo1 reporters were created with point mutations at the 5′ splice site (5′SSRibo1) or 3′ splice site (3′SSRibo1) respectively [23]. The mutant reporters were induced with doxycycline and the splicing intermediates detected using the primers shown in Figure 2 A. The merged time series for 3′SSRibo1 and 5′SSRibo1 are shown in Figure 2 C and 2 D respectively. As indicated by the error bars in Figure 2 C, the synthesis of lariat-exon2 in the mutant reporter varied between biological replicates, but technical error within each replicate remained at typical levels. The level of pre-mRNA measured in the modified reporters is greater than was observed for Ribo1. This may be attributed to changes in the rates of transcript synthesis, splicing step one or degradation, and modelling can help to resolve this question.

**Figure 3:**
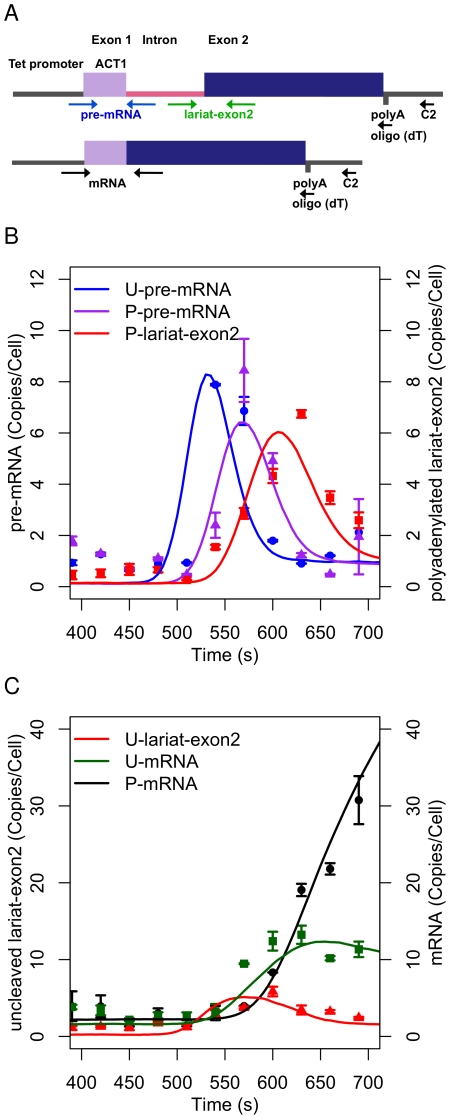
The 3′ cleavage and polyadenylation of Ribo1 measured by RT-qPCR. (A) Schematic of the Ribo1 gene. The positions of the alternative cDNA primers for uncleaved and cleaved/polyadenylated transcripts are shown by the C2 and oligo (dT) arrows respectively. (B) Points indicate uncleaved pre-mRNA (U-pre-mRNA), polyadenylated pre-mRNA (P-pre-mRNA) and polyadenylated lariat-exon2 (P-lariat-exon2) data. (C) Points indicate 3′ uncleaved lariat-exon2 (U-lariat-exon2), uncleaved mRNA (U-mRNA) and polyadenylated mRNA (P-mRNA) data. Error bars show the standard error of three biological replicates. Solid lines are model predictions.

For practical reasons, co-transcriptional splicing is defined here as splicing that is completed before the transcript has been released from the transcription complex by 3′ end cleavage. The data shown in Figure 2 were produced using a cDNA primer that hybridises to exon2 (at position C1 in Figure 2 A), which does not distinguish between transcripts that are cleaved and polyadenylated or uncleaved at the 3′ end. Therefore, in order to differentiate between these species and to estimate the rates for 3′ end formation, a modified 3′ cleavage assay used two alternative primers for cDNA synthesis from 3′ end sequences of Ribo1; oligo (dT), anneals to cleaved and polyadenylated transcripts, whereas primer C2 is complementary to a sequence downstream of the mapped 3′ end cleavage sites (Figure 3 A; [23]). By amplifying these cDNAs with specific primers for detection of pre-mRNA, lariat-exon2 and mRNA (Figure 3 A), uncleaved and cleaved/polyadenylated pre-mRNA, lariat-exon2 and mRNA were successfully distinguished in Expt 2 (Figure 3 B and 3 C).

The 3′ cleavage assay detected a sharp, transitory peak in uncleaved pre-mRNA at 540 s, followed by a similar peak in polyadenylated pre-mRNA 30 s later (Figure 3 B). This indicates pre-mRNA that is not spliced prior to 3′ end cleavage, i.e. is not spliced co-transcriptionally. However, the rapid accumulation of uncleaved mRNA between 540 and 600 s prior to detection of polyadenylated spliced mRNA at 600 s, clearly shows that co-transcriptional splicing occurs before post-transcriptional splicing. By formally modelling the splicing pathway, we aim to quantify the extent to which mature mRNA is derived from co-transcriptional splicing, and from post-transcriptional splicing respectively.

The reactions in the model must be enabled (switched on) progressively in order to explain the data. Following the induction of transcription by doxycycline, a burst of pre-mRNA is postulated to occur. At this time, splicing is not active, and additional transcripts are not initiated. These initial pre-mRNAs are cleaved and polyadenylated, and may then splice or degrade. This process explains the accumulation of pre-mRNA in Figure 2 B, and the peak in uncleaved pre-mRNA in the 3′ cleavage data in Figure 3 B. After a delay (defined by the rate 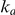), splicing steps 1 and 2 and the initiation of new transcripts start. This explains the drop in pre-mRNA in Figure 2 B, and the peak in polyadenylated pre-mRNA (Figure 3 B) as the activation of splicing removes these species also. Figure 1 in Text S1 illustrates the sequence of events.

### Alternative models of RNA processing steps

The proposition that there are advantages to modelling elongation in detail can be tested. Pathways that include 40 steps of elongation are compared with simpler pathway models where competition between elongation and splicing step one is represented by two reactions 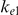 and 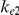 that have *APC* as the substrate and 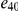 and 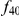 as the respective products. The proportion of co-transcriptional splicing can be calculated from these reaction rates and this proportion can be compared with that predicted for the 40 step model (as defined by equation 1 in Materials and Methods). The total time allocated to elongation can also be compared in the alternative models.

On completion of elongation, the RNA transcripts undergo 3′ end formation. This involves cleavage, polyadenylation and transcript release, and requires three multi-subunit factors [32]. Polyadenylation adds up to approximately 250 nucleotides to the end of the transcript. Hence, it is uncontroversial to model 3′ end formation as a multi-step process as many steps of maturation are clearly required. When fitting the splicing pathway to the Ribo1 data, a much better qualitative and quantitative fit is obtained when 3′ end maturation is modelled as a five-step process (each of the five steps has rate 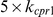) in comparison with a single step model. The characteristics of 3′ uncleaved spliced RNA also fit the data better by modelling 3′ end formation in this way. As shown in Figure 3 C, uncleaved mRNA quickly peaks towards its steady state of 10 copies/cell rather than making a slow progression to this level. Replacing the single step model with the 5 step model of 3′ end maturation (reactions 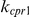 and 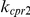) significantly improves the fit to the data. It is easily shown that a process of five steps, each at the same rate, has a kinetic response that differs significantly from that of a single step. (The distribution of waiting times follows a gamma distribution rather than an exponential distribution.) We do not aim to determine the exact number of steps, rather we aim to test whether a process of multiple steps of maturation or senescence provides a better quantitative and qualitative explanation than a single reaction. Henceforth, we assume that 5 steps constitute an adequate model of a multi-step process for the purpose of testing this hypothesis. Text S2 presents an analysis of Ribo1 degradation kinetics that further illustrates the approach.

Genetic studies have identified many splicing factors, but their impact on splicing kinetics *in-vivo* is difficult to quantify. These factors, and the five snRNPs, are not believed to be rate-limiting and have not been included in the model: We initially consider the kinetics of the splicing intermediates alone. However, we find once more that simple unimolecular models for steps one and two of splicing do not fit the data well. Consequently, we propose two alternative characterisations of the splicing reactions prior to steady state, and quantify the extent to which these models improve the fit of the pathway to the data.

The first alternative model of splicing we propose represents these processes as a sequence of several reactions that reflect the many known steps of spliceosome assembly. The precursors and products of multi-step processes show sharp transitions in their kinetics, as observed for pre-mRNA and lariat-exon2 in the experimental data. A multi-step model of this kind has been shown to explain fluorescence recovery after photobleaching (FRAP) data obtained from a splicing reporter in mammalian cells [33].

The second alternative explanation of the rapid processing of pre-mRNA and lariat-exon2 that we propose is based on the proposition that the splicing reactions are catalysed in a manner such that the propensity of the reaction increases on successive splicing events. It is necessary for the initial propensity to be low as otherwise no accumulation of splicing precursor or lariat intermediate would be observed, and for the propensity to increase to remove the accumulation rapidly. The reduction of uncleaved lariat-exon2 over the period of time when mRNA increases rapidly (600 s-700 s in Figure 3 C) may indicate such an increase in reaction propensity: the substrate decreases while the rate of increase of product remains constant. It therefore appears that step two of splicing may not be governed by first-order kinetics when it is co-transcriptional. The observations can be modelled by positive feedback in the splicing reaction. This requires the involvement of additional molecular species in the splicing reaction - the enzyme *Y* - a role that can be played by factors required for step two of splicing.

The following positive feedback mechanism has the property of increasing reaction propensity: Let the enzyme *Y* have an initial copy number of 1, and increment the copy number on each splicing event to effectively increase the propensity. The enzyme contributes to the reaction propensity according to the formula for bimolecular reactions (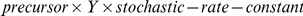). The positive feedback model is proposed for the kinetics of high rates of induction prior to steady state.

### Pathway comparison

Due to the uncertainties in pathway structure, and parameter identifiability and estimation, it is of considerable value to explore multiple models of a biological system [34]. The goodness of fit to the experimental data of eight versions of the RNA processing pathway is compared in Table 1. The alternative pathways are distinguished by their representation of elongation, of co-transcriptional splicing step two, and of post-transcriptional splicing steps one and two. Elongation is modelled either as a single step or as 40 steps, as described above. The alternative models considered for the splicing reactions are: a single step, multiple steps (each at the same rate) or positive feedback. It is important to consider each pathway as a whole as the goodness of fit for each observed species is dependent on the reactions that act directly on the observed species, and those that act on its precursors and thereby shape the kinetics of the precursors. Table 1 defines each pathway and lists the AIC scores obtained using the optimal parameters (see Table 1 in Text S1 for the parameter values). Note that pathway slowromancap VIII makes the simplest assumptions about elongation and splicing steps, namely that they are single-step unimolecular reactions, and that the poor fit of this pathway to the data motivates the search for alternative models.

**Table 1:** Comparison of pathways I-VIII using optimal parameters by AIC.

Pathway	Property			Total AIC	Akaike wt.
	Co	Po	El		
I	F	M	M	0	0.845
II	F	M	S	40.9	1.09e-9
III	F	F	M	3.43	0.152
IV	F	F	S	31.2	1.40e-7
V	M	M	M	16.5	2.20e-4
VI	M	M	S	36.1	1.25e-8
VII	S	S	M	11.7	2.41e-3
VIII	S	S	S	28.4	5.65e-7

The alternative pathways are distinguished by the representation of co-transcriptional splicing step 2 (column Co) as a reaction that involves feedback (F), multiple steps (M) or a single step (S); the representation of post-transcriptional splicing steps 1 and 2 (column Po) as a F, M or S reaction; and their representation of elongation (column El) as a M or S process. Total AIC is calculated from all pathway model residuals in all species, and normalised by subtracting the AIC for pathway I. Akaike weights 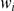 are calculated from total AIC and represent the normalised likelihood of each of the eight pathways (see Materials and Methods, and Table 1 in Text S1 for parameter values).

Pathway parameters were optimised by a simulated annealing algorithm (see Materials and Methods; [35]) that identified the best fit between each pathway and the nine data series obtained for Ribo1 (those plotted in Figure 2 B, 3 B and 3 C). The total AIC (defined in Materials and Methods) is calculated from the combined residuals from all species/experiments. All data and pathway models are provided as files in Dataset S1. An executable version of the Dizzy simulator [36] is also provided to allow the models to be executed.

**Figure 4:**
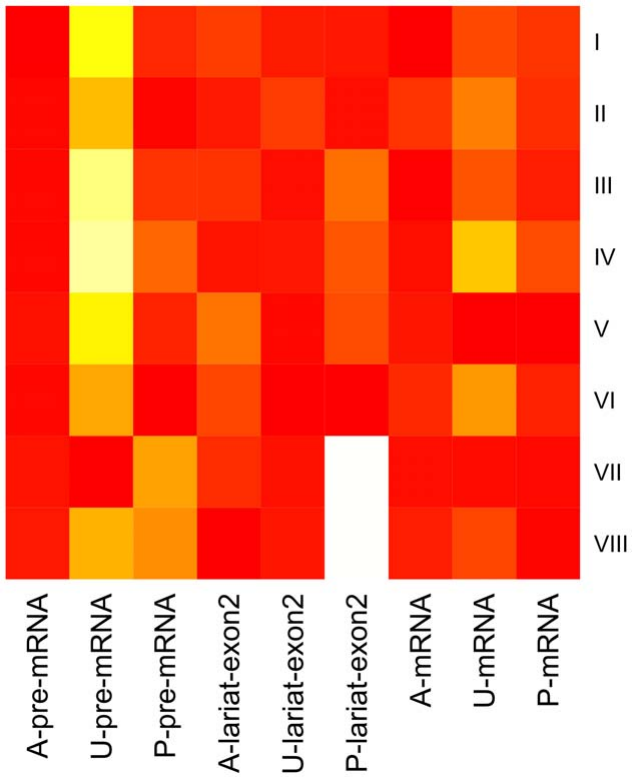
A heat map of AIC scores for pre-mRNA, lariat-exon2 and mRNA. A- indicates the AIC score for all species in Expt 1 (uncleaved and polyadenylated); U- AIC for uncleaved species in Expt 2; and P- AIC for polyadenylated species in Expt 2. The spectrum red to white reflects the lowest (best) to highest (worst) AIC score calculated for each species using the optimal parameter values for each pathway (rows I-VIII). Each column is normalised to have a minimum value of 0 by subtracting the minimum AIC score in the column from each entry.

The AIC scores for pre-mRNA, lariat-exon2 and mRNA are represented separately in the columns of the heat map in Figure 4. It is apparent from the A-pre-mRNA column that all pathways fit well to the pre-mRNA data in Expt 1, and fit to a comparable extent. The majority of pathways also fit the mature mRNA data well (A-mRNA and P-mRNA columns). Pathways I-IV can be optimised to the lariat-exon2 data simultaneously. In contrast, pathways V-VIII have a poor fit to one or more of the lariat-exon2 species. Pathway I has the best overall AIC as a result of fitting the nine data series most consistently.

Pathways I-IV incorporate the feedback mechanism for co-transcriptional splicing step two and this feature correlates with a good fit (low AIC) for all lariat-exon2 species. Within these pathways, a multi-step representation of post-transcriptional splicing, combined with a multi-step representation of elongation has the best overall score (pathway I). Feedback in post-transcriptional splicing, combined with a multi-step representation of elongation also explains the data well (III). Pathway VII is ranked in third place, failing to explain the polyadenylated lariat-exon2 data in Expt 2 (as indicated by the white cell in row VII, column P-lariat-exon2 in Figure 4). The predictions of the pathways for each of the nine species measured in Expt 1 and 2 are plotted in Figure 2 in Text S1. Important qualitative differences between the pathways can be seen in these graphs.

### Elongation and 3′ end maturation are multi-step processes

Pathways with a single elongation step require an initiation rate of 0.4, and a rate for elongation 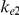 of 0.4–0.54, giving an implausible time of 2–3 s for the elongation of a 1240 nucleotide gene. As a consequence of defining a more realistic elongation time, pathways with a multi-step representation of elongation typically fit the data better, see Table 1.

In pathway I, pre-mRNA 3′ end maturation takes 35 s and uncleaved 3′ end mRNA maturation takes 49 s using the measure of the time taken for the sum of intermediate species undergoing the five steps of 3′ end processing to reduce by half (an equivalent to the half life of a single step reaction).

### Splicing is predominantly co-transcriptional for Ribo1

The completion of splicing co-transcriptionally in yeast has been a topic of debate. Genome-wide ChIP studies indicated that co-transcriptional spliceosome assembly may not have time to occur if the 3′ exon is short [28]. More recent studies provide evidence for polymerase pausing 3′ of introns, suggesting a mechanism to slow transcription, allowing more time for splicing [10], [11]. With Ribo1 we observe that the initial burst of 3′ uncleaved pre-mRNA is not spliced before it is 3′ end cleaved, as shown by the successive blue and purple peaks in Figure 3 B, and it may undergo post-transcriptional splicing. After this initial burst, the majority of transcripts splice co-transcriptionally, as seen by the accumulation of uncleaved lariat-exon2 and uncleaved mRNA prior to cleaved/polyadenylated mRNA (red, green and black, respectively in Figure 3 C). Optimisation of pathway I computes 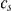 to be 11.39, and by substituting this value into equation 1 (see Materials and Methods) it follows that 12% of Ribo1 RNA transcripts splice post-transcriptionally, and 88% of transcripts splice co-transcriptionally. Values for 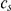 in pathways III, V and VII imply the same proportion of co-transcriptional splicing, as do the values of 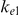 and 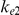 in the four remaining pathways where the proportion of co-transcriptional splicing is approximately 85%.

### The second step of splicing is governed by positive feedback when co-transcriptional

Pathways I-IV show a good qualitative fit to the uncleaved lariat-exon2 data (see Figure 2E in Text S1). All four pathways specify a positive feedback mechanism for 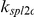 with estimated rate constants 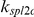 in the range 0.0061–0.0068 (see Table 1 in Text S1). In pathway I , the half life of this reaction is 110 s for the first transcript to splice, and, with feedback, the half life reduces to 5.5 s at 670 s after induction. As, initially, the half life is much greater than the time to transcribe exon 2 (approximately 11 s), the decision to model the second step of splicing as a process that occurs after elongation is justified.

The feedback mechanism may be a result of the disassembly and recycling of the snRNPs of the spliceosome for subsequent rounds of splicing [37]. It has been proposed that the branchpoint binding protein (BBP) and Mud2 are recycled between two steps in pre-spliceosome assembly: BBP is released during or after the second step and efficiently recycled to promote the first [38]. The finding that snRNPs do not assemble on a nascent transcript in response to a signal, but move randomly [22], does not preclude them impacting on splicing kinetics in a transcription-dependent manner through an influence on rates of spliceosome assembly, disassembly or recycling. Maintenance of the transient Cajal body (responsible for the maturation of snRNPs) requires continuous recycling of pre-existing snRNPs after each round of spliceosome assembly [22], and may therefore be indirectly dependent on transcription when splicing is co-transcriptional. If recycling mechanisms existed for second step factors, increasing the effective second step reaction rate, this could explain the peak and dip in uncleaved lariat-exon2 in Expt 2. The allosteric effects of second step splicing factors would provide an alternative explanation.

### Post-transcriptional splicing has multi-step characteristics

Pathways I and III specify a multi-step representation of elongation and feedback in co-transcriptional splicing. They account for 99% of the probability mass available in the Akaike weight analysis (see Table 1). These two pathways differ on the post-transcriptional splicing mechanism: a multi-step representation is more probable (P = 0.845) but a feedback mechanism cannot be ruled out (P = 0.152). As pathway I has a better fit to the polyadenylated pre-mRNA and polyadenylated lariat-exon2 data (the precursor and products of post-transcriptional splicing), we tentatively conclude that post-transcriptional splicing has multi-step characteristics. The difficulty in modelling the post-transcriptional splicing process lies in its transient activation. The characteristic features of the feedback mechanism are not clearly revealed. For a multi-step model, the times for the sum of intermediate species undergoing splicing to reduce by half are 34 s for step one and 36 s for step two of splicing.

### A mutation at the 3′ splice site reduces the probability of co-transcriptional splicing

The 3′SSRibo1 data are explained by a variation of the transcription and splicing pathway where step one of splicing can be co-transcriptional or post-transcriptional (as in the full pathway), but where the lariat-exon2 species goes through the five-step cleavage process (at rate 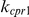) instead of step two of splicing (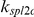 = 0 and 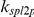 = 0). The pathway used to explain the 5′SSRibo1 data has no co-transcriptional splicing path (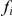 cannot be reached), and no post-transcriptional splicing can occur (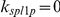).

The induction of the 3′SSRibo1 reporter (Figure 2 C) shows a greater accumulation of pre-mRNA than observed for Ribo1. The lariat-exon2 product is not spliced, but accumulates and is subject to degradation. The data can be explained by pathway I using the 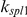 rate inferred for Expt 1 and 2. However, to predict the pre-mRNA response 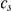 is increased to 30, 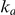 is reduced to 0.015, and 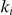 is reduced to 0.175. The probability of step 1 occurring co-transcriptionally is therefore reduced to 56% compared with 88% in Ribo1, the time taken for splicing to become active increases two fold, and the rate for the initiation of transcription reduces to 70% of the rate in Ribo1. The prediction for lariat-exon2 is greater than observed, and this may indicate that 3′ end maturation and/or lariat-exon2 degradation pathways differ in the mutant reporter.

The induction of 5′SSRibo1 (Figure 2 D) shows that pre-mRNA accumulates and does not splice. The response can be explained by further reduction in 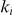 to 0.1, that is, 40% of the rate in Ribo1.

The induction of 3′SSRibo1 was repeated using the primers of Expt 2 in order to validate the finding that the probability of co-transcriptional splicing is reduced. The new data are shown in Figure 3A in Text S1. The pathway model predicts only a slow removal of the accumulating uncleaved pre-mRNA (and consequently of polyadenylated pre-mRNA) that is consistent with the new data. In contrast, the large reduction in pre-mRNA that is predicted when the rate for 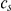 is 11.39 (as inferred for Ribo1) does not fit the new data, see Figure 3B in Text S1.

The overestimation of lariat-exon2 by the model (Figure 2 C) might be explained by a significant underestimation of the degradation rate for this species. This rate has been determined in the 3′SSRibo1 ‘OFF’ strain where transcription is halted by doxycycline, see Text S2. (A second experiment using alternative primers confirms this result [23].) Alternatively, the assumption made when modelling 3SSRibo1 that uncleaved lariat-exon2 would be able to complete 3′ end maturation and contribute to the total population of polyadenylated lariat-exon2 may be incorrect. Modelling shows that polyadenylated lariat-exon2 may be the product of polyadenylated pre-mRNA alone, with no contribution from the co-transcriptional pathway.

## Discussion

Despite the biochemical and genetic evidence for multiple steps in the cycle of splicing events, previous *in vivo* studies of mRNA splicing kinetics have revealed simple first-order monomolecular reactions that exclude the action of a catalyst. The allosteric cascade is yet to be revealed at the systems level, either in terms of the existence of multiple steps, or the impact of enzyme kinetics, and we argue that this is due to the course-graining phenomena associated with stochastic processes [39] and to the lack of experimental quantification of mRNA and its precursors.

Using rapid sampling of cultures, combined with RT-qPCR assays that detect the intermediates and products of the splicing reaction in a way that permits quantitative comparisons between different RNA species and between different cultures, we are able to present kinetic data with an unprecedented level of resolution, monitoring pre-mRNA production, the two steps of splicing and 3′ end processing of a reporter transcript in yeast. Our data cannot be explained satisfactorily by single-step unimolecular splicing reactions. We conclude that a systems model of transcription and splicing must distinguish the two steps of splicing, account for their occurrence co- and post-transcriptionally, represent spliceosome assembly, and include the action of an additional partner in the splicing reactions, as we find evidence in the data for each of these processes.

While developing the model, we considered including a transition from uncleaved lariat-exon2 to polyadenylated lariat-exon2, which would permit pre-mRNAs that have already undergone the first step of splicing co-transcriptionally to undergo 3′ end maturation. However, when this transition was added to model I, the AIC was found to increase by 1.4 (after optimisation), meaning model I fits the data better without the additional transition. The proposed transition occurs very slowly, and consequently rarely, does not assist modelling the data, and, therefore, was excluded from the models we analysed further.

The model proposed here specifies that pre-mRNAs that have already undergone the first step of splicing co-transcriptionally will be fully spliced co-transcriptionally prior to 3′ end cleavage. This is in contrast with the mammalian model proposed in [40] where splicing is completed after 3′ cleavage (in HeLa nuclear extracts). Both models stipulate that partially-spliced transcripts are not released from the DNA, and both allow for a post-transcriptional splicing pathway. Our model is consistent with the recycling of splicing factors [3]. Recycling of BBP and Mud2 has been proposed for pre-spliceosome formation [38], and similar mechanisms may exist for subsequent spliceosome assembly steps. Alternatively, it has been proposed that an increase in the local concentration of splicing factors is linked to transcription via the C-terminal domain of Pol II [15]. Nuclear speckles may also have a role in keeping spliceosomal components concentrated near nascent transcripts [37]. Cooperativity in the interaction of splicing factors with the spliceosome or with the nascent pre-mRNA may also contribute to the kinetics of co-transcriptional splicing.

Addressing the interdependency between RNA processing steps, modelling indicates that mutations at the 3′ and 5′ splice sites reduce the rate of initiation of transcription, and, in the 3′SS mutant, reduce the probability of step one of splicing occurring co-transcriptionally. Quantitative analysis of the mutant data requires establishing a parameterised model for the ‘wild type’ in order to define and test the alternative explanations of the differences observed.

A half life for splicing in HeLa cell nuclear extracts of 23 min (splicing rate of 0.03/min) has been reported [15]. *In vivo* half-lives of 6–12 min have been reported in mammalian cells [41], as have estimates of 5–10 min for the completion of splicing after intron synthesis [42]. Half lives for splicing in the range 0.4–7.5 min have also been reported for the splicing of introns in mammalian cells [43]. The inferred rates for post-transcriptional splicing in Ribo1 equate to half lives of 0.6 min for each of steps one and two, and are at the faster end of the spectrum reported in [43]. On the co-transcriptional pathway, splicing step one is concurrent with the transcription of the 800 bases from the branchsite until the polyA site (taking approximately 11 s). Co-transcriptional step two occurs with a half life of 110 s for the first transcript, and, with feedback, the half life reduces to 5.5 s at 670 s after induction. Therefore co-transcriptional splicing is the more efficient pathway under the high induction conditions studied here.

This study proposes a mechanistic kinetic model that represents some of the complexity and flexibility of the splicing pathway that is known from biochemical and genetic studies [3]. Co-transcriptional splicing is evident in the data, and modelling shows that this pathway may be activated after a delay. Furthermore, the second step of splicing benefits from positive feedback when co-transcriptional. These could be explained by the coordination of splicing factor recruitment/recycling with transcription, possibly facilitated by polymerase pausing [10], [11] and/or dynamic chromatin modification [9], [44], [45].

## Materials and Methods

### Strains and RT-qPCR

To analyse the transcription, splicing, degradation and 3′ end formation of yeast pre-mRNA, the Ribo1 reporter was integrated into the yeast genome at the *his3* locus. The reporter is based on a hybrid *ACT1/PGK1* gene [31], modified as described in [23] by inserting two copies of the 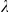 boxB sequence (57 bp each) in the *ACT1* intron, enabling it to be readily distinguished by RT-qPCR from the endogenous *ACT1* intron without affecting splicing.

Primer pairs were created to measure the unspliced pre-mRNA (5′ primer upstream of ATG, 3′ primer over the exon 1 - intron junction), the lariat-exon2 intermediate (5′ primer upstream of 3′end of intron, 3′ primer over exon 2; the pre-mRNA level was subtracted from this measurement) and the spliced mRNA (5′ primer upstream of ATG, 3′ primer over exon 2). Measurements of mRNA in copies per cell, averaged over a population, were obtained by carefully quantifying the efficiency of cell lysis, recovery of RNA, reverse transcription and qPCR. For full details see [23].

### Modelling the RT-qPCR signal

The first step of post-transcriptional splicing, and all transitions to the 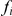 path, decrease pre-mRNA and increase lariat-exon2. The second step of splicing decreases lariat-exon2 and increases spliced mRNA, according to the pathway. All species in the pathway, with the exception of the excised intron product of step two, are measurable by RT-qPCR, provided that they extend beyond the position of the cDNA primer. Splicing events on transcripts that have not been elongated to the cDNA point are not detected until this sequence is transcribed, and the calculation of RT-qPCR signal intensity from the species in the pathway reflects this. For example, the (simulated) pre-mRNA signal is not incremented until the 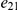 species is incremented, despite the PCR primers for pre-mRNA being located several hundred bases upstream.

Considering a single Pol II complex (and ignoring the effect of other Pol IIs on its movement), the probability of transitioning from states 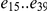 to the co-transcriptional path is simply calculated from the elongation rate and the transition rate. This choice can be made 25 times, allowing the probability of the Pol II exiting on the post-transcriptional pathway to be estimated independently of 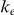 by:
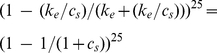
(1)


Unless otherwise stated, reaction rates are expressed as the probability density per unit time, per distinct combination of reactant molecules. Where there is a single reactant species, the number of distinct combinations is just the population of reactants. The half life is the time a molecular species takes to reduce by half, and is computed for unimolecular reactions by 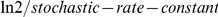 in units of seconds.

### Pathway optimisation and comparison

Pathway models were optimised by the simulated annealing algorithm specified in [35] (see Figure 1). Following [35], the error *E* is defined by equation 2 where *S* is a time series simulated from a pathway model, *D* is the observed data, *n* is the number of time points and *d* the number of dependent variables (the dimension of 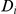 and 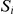 is *d*).
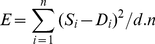
(2)On each iteration of the algorithm, each parameter 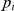 is assigned a new value (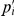) and the error for the new set of parameters (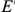) is calculated from a simulation of the model using the updated parameter set. The new parameter value is always accepted if 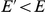, otherwise it is accepted with probability 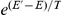, where *T* is the current temperature and *E* is the error of the current parameter set. The new parameter value is generated from the current value by adding a normally-distributed random value. We define the scale constant *k* in equation 3 using the error of a set of parameter values that are given as input at the start of optimisation (these must provide an approximate fit to the data), and then update each parameter value according to equation 4, where N(0,1) is a normally-distributed random value (mean 0, standard deviation 1) and 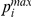 and 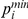 are the maximum and minimum values respectively that 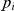 is allowed to take. See [35] for further details.
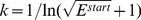
(3)

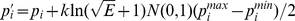
(4)


The Akaike information criterion (AIC; eqn. 5) was used to assess the fit between a time series *S* simulated from a pathway model of *k* optimised parameters, a data set *D* of *n* values [46]. Assuming normally distributed errors, AIC can be computed from the model residuals (eqn. 6) [47]. The values for total AIC incorporate the *2 k* penalty for the number of parameters optimised.
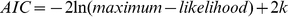
(5)

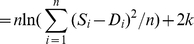
(6)When comparing *m* pathway models, the Akaike weight *w* 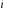 of model *i* can be defined in terms of the relative likelihood 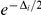, where 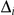 is the difference between the AIC for model *i* and the AIC of the best model [47]. Akaike weights computed by equations 7 and 8 are listed in Table 1 .
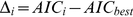
(7)

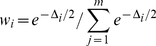
(8)


## Supporting Information

Dataset S1 **Models I-VIII, experimental data and simulator scripts.**
(BZ2)

Text S1 **Supplementary figures and model parameters.** Supplementary figures on model fitting, and a table of optimal parameters for models I-VIII.
(PDF)

Text S2 **The kinetics of multi-step processes.** Analysis of the degradation of pre-mRNA and lariat-exon2 of 3′ and 5′ splice site mutants of the Ribo1 reporter.
(PDF)
