# Supplementary material for: Modelling Reveals Kinetic Advantages of Co-Transcriptional Splicing
Source: PLoS Comput Biol. 2011 Oct 13;7(10):e1002215. doi: 10.1371/journal.pcbi.1002215 (PMC3192812; doi:10.1371/journal.pcbi.1002215)
Supplement: Text S1 — Supplementary figures and model parameters. Supplementary figures on model fitting, and a table of optimal parameters for models I-VIII. (PDF) [file pcbi.1002215.s002.pdf]

Modelling reveals kinetic advantages of co-transcriptional splicing - Supplementary Text 1

Stuart Aitken[1*], Ross D. Alexander[1,2], Jean D. Beggs[1,2]
1 Centre for Systems Biology, University of Edinburgh, Edinburgh, UK
2 Wellcome Trust Centre for Cell Biology, University of Edinburgh, Edinburgh, UK
∗ E-mail: s.aitken@ed.ac.uk

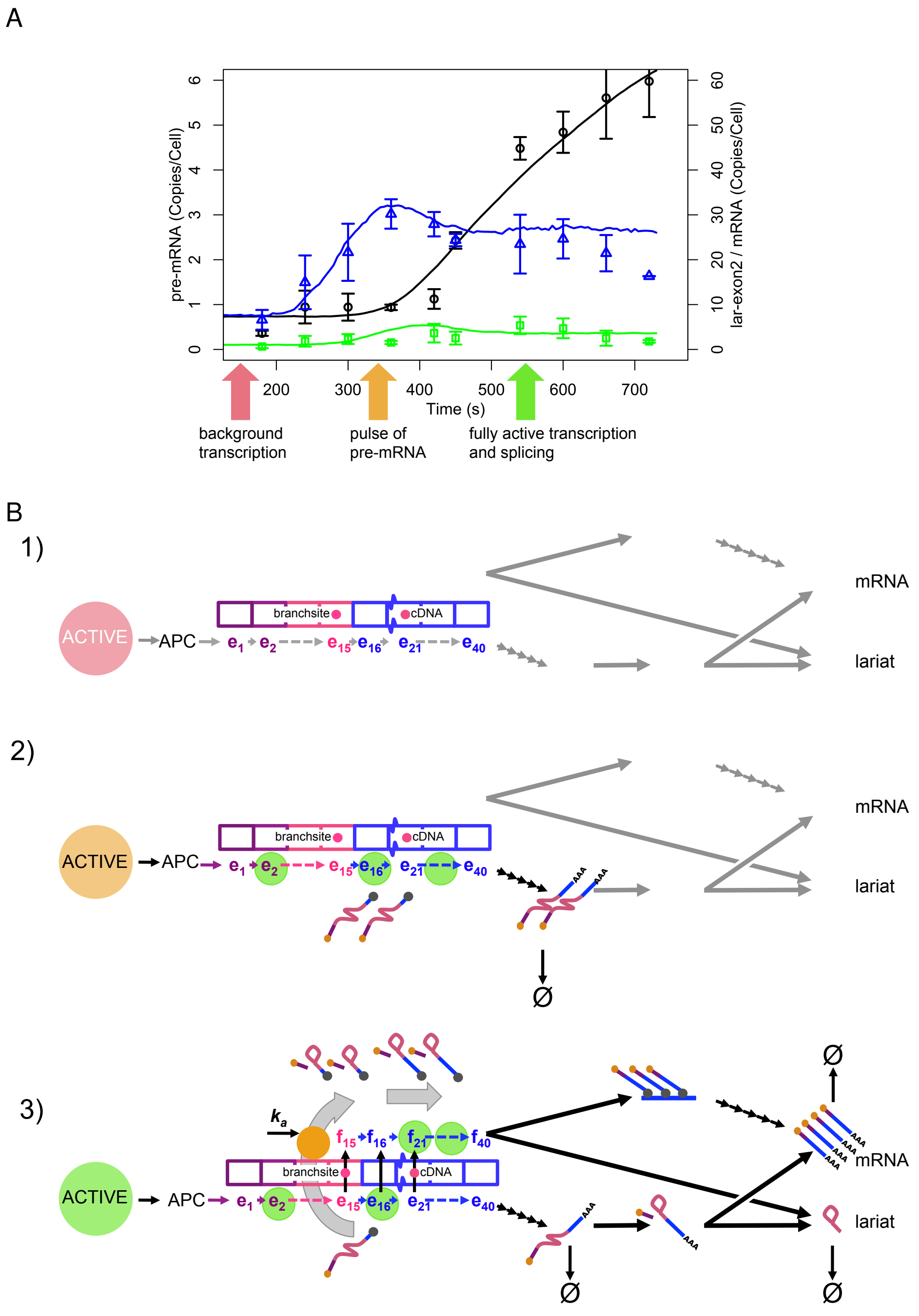

Figure 1. The sequence of Ribo1 activation. (**A**) Events in Expt 1. (**B**) 1) Background transcription only. The predictions are based on the steady-state levels of the species (a proportion of cells are active, and establish an average of approximately 10 copies/cell of mRNA across the population). 2) A pulse of transcription occurs on activation of Ribo1. Pre-mRNA accumulates as splicing remains inactive. 3) The initiation of new transcripts, and splicing steps 1 and 2 are active. Reactions that are inactive are shown in grey. The degree of activation of the alternative splicing pathways is indicated approximately by the number of transcripts.

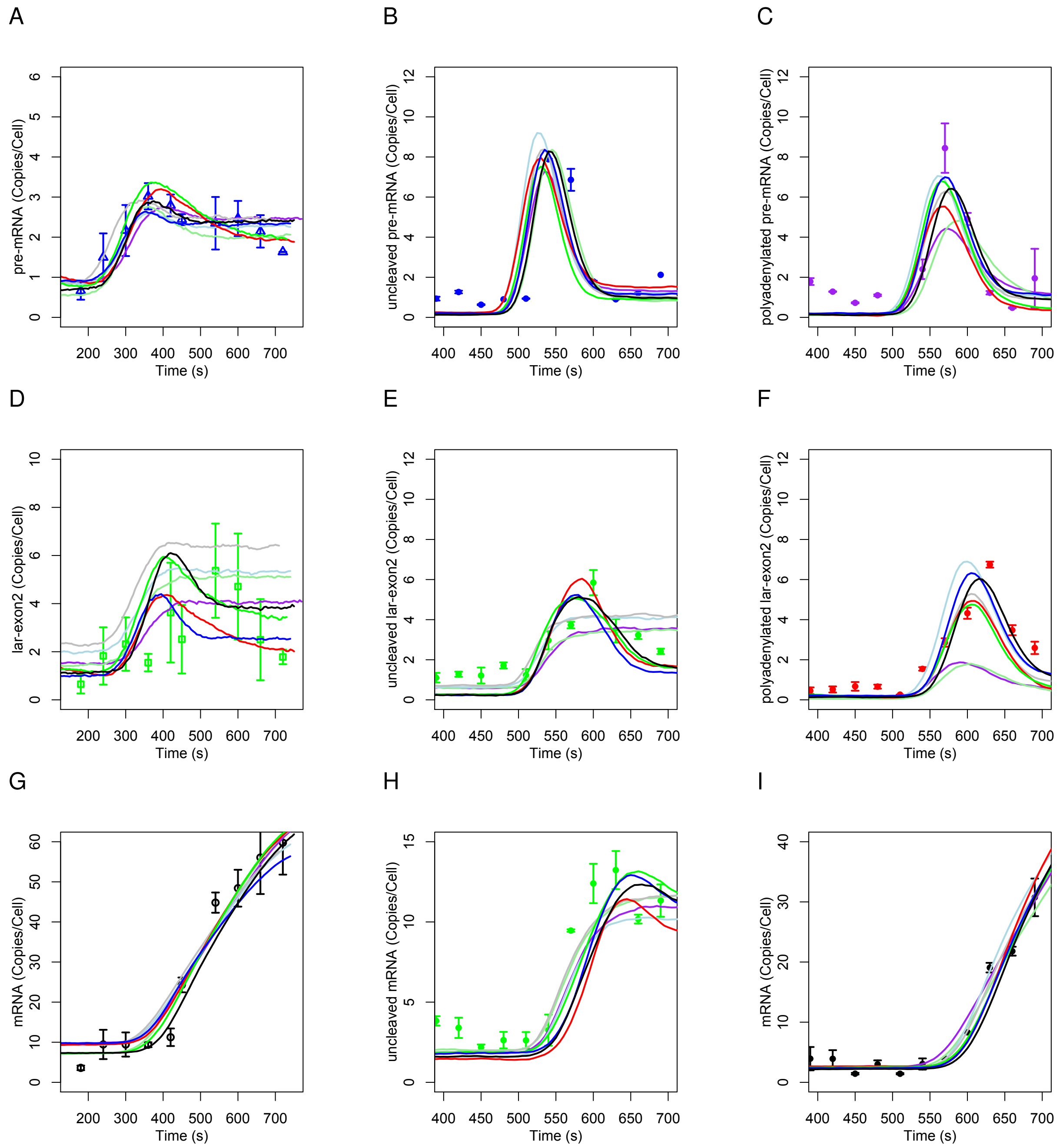

Figure 2. Simulations of pathways I-VIII for optimal parameters in Expt 1 and 2. (**A**) pre-mRNA (Expt 1); (**B**) uncleaved pre-mRNA (Expt 2); (**C**) polyadenylated pre-mRNA (Expt 2); (**D**) lariat-exon2 (Expt 1); (**E**) uncleaved lariat-exon2 (Expt 2); (**F**) polyadenylated lariat-exon2 (Expt 2); (**G**) mRNA (Expt 1); (**H**) uncleaved mRNA (Expt 2); (**I**) polyadenylated mRNA (Expt 2); The colour coding in each figure is: pathway I (black); II (blue); III (green); IV (red); V (grey); VI (light blue); VII (light green); VIII (purple);

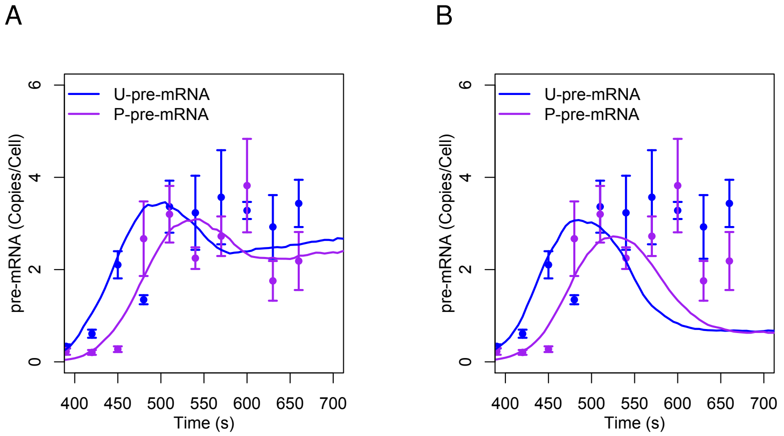

Figure 3. The response of the 3'SSRibo1 reporter to doxycycline. The cDNA primers for uncleaved and cleaved/polyadenylated transcripts are those used in Expt 2 (see Figure 3A). (**A**) Points indicate uncleaved pre-mRNA (U-pre-mRNA) and polyadenylated pre-mRNA (P-pre-mRNA) data. Error bars show the standard error of two biological replicates. In order to match the total pre-mRNA in Figure 2C, and thereby make predictions using the same pathway model parameters as in Figure 2C, the data is scaled by a factor that equates the peak value of uncleaved pre-mRNA + polyadenylated pre-mRNA observed in this experiment to that of Figure 2C (7.1 copies/cell). Solid lines are pathway predictions using c_s=30, k_a =0.015, k_i= 0.175, otherwise the optimal parameters are used. (**B**) Uncleaved pre-mRNA and polyadenylated pre-mRNA predicted for the parameter values used in (A) except c_s=11.39. The increased probability of co-transcriptional splicing does not fit the data.

Pathway	Optimal parameter values							
	c_s	k_{e1} k_{e2}	k_{cpr1}	k_{cpr2}	k_{spl1p}	k_{spl2p}	k_{spl2c}	$k_{a,Expt1}$	$k_{a,Expt2}$
I	11.39		0.0266	0.0189	0.0277	0.0257	0.0063[§]	0.0257	0.0340
II		0.0728 0.3950	0.0290	0.0171	0.0284	0.0274	0.0068[§]	0.0323	0.0454
III	11.94		0.0307	0.0175	0.0051[§]	0.0061[§†]	0.0061[§†]	0.0336	0.0336
IV		0.0949 0.5211	0.0245	0.0228	0.0064[§]	0.0062[§†]	0.0062[§†]	0.0371	0.0371
V	11.37		0.0252	0.0172	0.0276	0.0294	0.0470	0.0257	0.0340
VI		0.0740 0.4459	0.0270	0.0190	0.0294	0.0257	0.0473	0.0359	0.0377
VII	10.24		0.0238	0.0175	0.0243	0.0589[†]	0.0589[†]	0.0359	0.0341
VIII		0.0934 0.5415	0.0267	0.0192	0.0281	0.0594[†]	0.0594[†]	0.0388	0.0451

Table 1. Optimal parameter values for pathways I-VIII obtained by Simulated Annealing. The probability of co-transcriptional splicing step 1 is determined by either c_s or k_{e1} and k_{e2} according to the model of elongation. The k_a parameter takes different values when fitting to Expt 1 ($k_{a,Expt1}$) and Expt 2 ($k_{a,Expt2}$).
Key: § a feedback model applies; † co-transcriptional and post-transcriptional splicing rates take the same value.
