# Supplementary material for: Modelling Reveals Kinetic Advantages of Co-Transcriptional Splicing
Source: PLoS Comput Biol. 2011 Oct 13;7(10):e1002215. doi: 10.1371/journal.pcbi.1002215 (PMC3192812; doi:10.1371/journal.pcbi.1002215)
Supplement: Text S2 — The kinetics of multi-step processes. Analysis of the degradation of pre-mRNA and lariat-exon2 of 3′ and 5′ splice site mutants of the Ribo1 reporter. (PDF) [file pcbi.1002215.s003.pdf]

Modelling reveals kinetic advantages of co-transcriptional splicing - Supplementary Text 2

Stuart Aitken[1*], Ross D. Alexander[1,2], Jean D. Beggs[1,2]
1 Centre for Systems Biology, University of Edinburgh, Edinburgh, UK
2 Wellcome Trust Centre for Cell Biology, University of Edinburgh, Edinburgh, UK
∗ E-mail: s.aitken@ed.ac.uk

Contents

Kinetics of multi-step processes **1**

Supplementary References **2**

Supplementary Tables **4**

Kinetics of multi-step processes

The degradation of Ribo1 pre-mRNA was measured in an "OFF strain" of 5'SSRibo1 where transcription can be halted by doxycycline (Alexander *et al.*, 2010), see Figure 1A. Similarly, the degradation of lariat-exon2 was measured in an OFF strain of 3'SSRibo1 (Alexander *et al.*, 2010), see Figure 1B.

Degradation can be modelled as a single reaction that depletes the population of species C from an initially constant level (model A). Alternatively, depletion can be modelled as occurring in multiple steps that produce a series of intermediates I_1 .. I_n (model B). In both cases, we can consider the corresponding accumulation of species D: the degraded molecules derived from C. These models are defined and solved below.

(A) single step of depletion

$$dC/dt = -\alpha C \quad (1)$$
$$dD_1/dt = \alpha C \quad (2)$$
$$C = C_0 e^{-\alpha t} \quad (3)$$
$$D_1 = C_0(1 - e^{-\alpha t}) \quad (4)$$

(B) multiple steps of depletion

$$dC/dt = -\alpha C \quad (5)$$
$$dI_1/dt = \alpha C - \alpha I_1 \quad (6)$$
$$dI_i/dt = \alpha I_{i-1} - \alpha I_i \quad (7)$$
$$dD_{n+1}/dt = \alpha I_n \quad (8)$$
$$C = C_0 e^{-\alpha t} \quad (9)$$
$$I_n = C_0 \alpha^n t^n e^{-\alpha t} \quad (10)$$
$$D_{n+1} = C_0 - C_0 e^{-\alpha t} - \sum_{k=1}^{n} C_0 \alpha^n t^n e^{-\alpha t}/n! \quad (11)$$

In model B, we might choose to define all intermediate species I_i as still being instances of C as they have not yet completed the process of becoming D. This is relevant to the degradation of mRNA as partially-deadenylated

mRNA remains competent for translation (if at a reduced rate of transcription initiation (Goldstrohm and Wickens, 2008)).

Based on the ODE models of degradation as a 1 step (model A), or a 2 or 3 step process (model B), the following functions were optimised to the data using a nonlinear least squares method implemented in R (R Foundation). We assume the intermediate species contribute to the precursor C ($C_{n+1} = C_0 - D_{n+1}$). In addition to the rate parameter α, optimal values for the scaling parameter β and offset γ were also identified.

$$C_1 = \gamma + \beta e^{-\alpha t} \quad (12)$$

$$C_2 = \gamma + \beta(e^{-\alpha t} + \alpha t e^{-\alpha t}) \quad (13)$$

$$C_3 = \gamma + \beta(e^{-\alpha t} + \alpha t e^{-\alpha t} + \alpha^2 t^2 e^{-\alpha t}/2) \quad (14)$$

The alternative model predictions are shown in Figure 1, and the AIC scores and Akaike weights for each model are listed in Table 1. The Akaike weights, w_i, can be interpreted as the probability of model i, given the set of three candidate models under consideration. For lariat-exon2, the 2 step model is the most probable (P=0.59), but the simple exponential decay model (1 step) is also a candidate (P=0.24). For pre-mRNA, the 3 step model has probability 0.92, leaving only a probability of 0.08 that one of the other models applies. Therefore, there is considerable evidence for multiple steps in pre-mRNA degradation. Degradation is known to be a multi-step process, and has been modelled in detail (Cao and Parker, 2003). The optimal model parameters are listed in Table 2.

References

Alexander, R., Barrass, D., Dicht, B., Kos, M., Obtulowicz, T., Robert, M.-C., Korper, M., Karkusiewicz, I., Mariconti, L., Tollervey, D., Dichtl, B., Kufel, J., Bertrand, E., and Beggs, J. (2010). Ribosys, a high resolution, quantitative approach to measure the in vivo kinetics of pre-mRNA splicing and 3' end processing in *saccharomyces cerevisiae*. *RNA*, **16**, 2570–2580.

Cao, D. and Parker, R. (2003). Computational modeling and experimental analysis of nonsense-mediated decay in yeast. *Cell*, **113**(4), 533–545.

Goldstrohm, A. C. and Wickens, M. (2008). Multifunctional deadenylase complexes diversify mRNA control. *Nat Rev Mol Cell Biol*, **9**(4), 337–344.

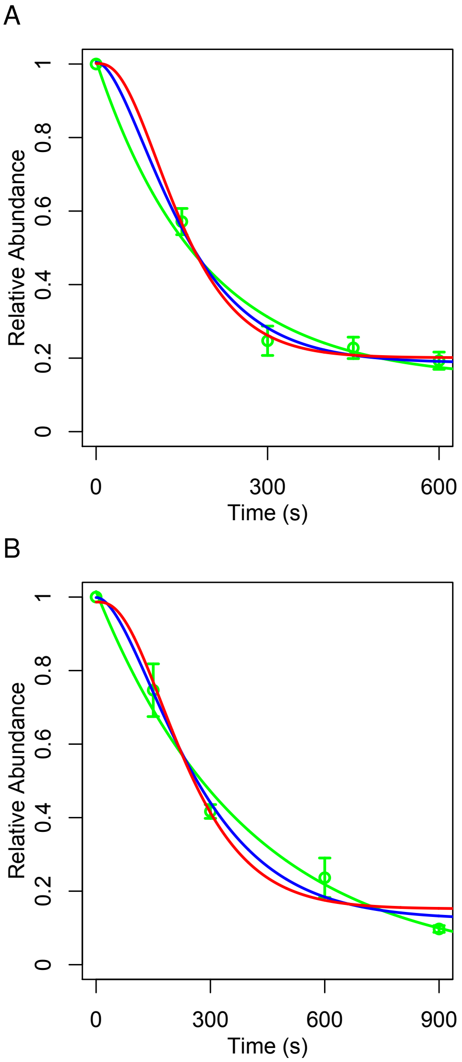

Figure 1. The degradation of 5'SSRibo1 and 3'SSRibo1 products. (**A**) Degradation of unspliced pre-mRNA (5'SSRibo1). (**B**) Degradation of lariat-exon2 (3'SSRibo1). Symbols indicate data. Error bars show the standard error of three biological replicates. Solid lines are model predictions: 1 step model (green); 2 step model (blue); 3 step model (red).

Model	pre-mRNA		lariat-exon2	
	AIC	Akaike wt.	AIC	Akaike wt.
1 step	-10.8	0.003	-11.0	0.24
2 step	-17.0	0.074	-12.8	0.59
3 step	-22.1	0.923	-10.3	0.17

Table 1. Comparison of degradation models for 5'SSRibo1 and 3'SSRibo1 products. Akaike weights represent the normalised likelihood of each of the three models (see Materials and Methods).

Data	Model	γ	β	α (half-life min.)
pre-mRNA	1 step	0.141266	0.867559	0.005415 (2.1)
pre-mRNA	2 step	0.18691	0.81739	0.01231 (1.9)
pre-mRNA	3 step	0.20105	0.80033	0.01904 (1.8)
lariat-exon2	1 step	-0.004757	1.019712	0.002534 (4.6)
lariat-exon2	2 step	0.122608	0.876035	0.007215 (3.2)
lariat-exon2	3 step	0.15184	0.83459	0.01180 (2.9)

Table 2. Optimal parameter values for 5'SSRibo1 and 3'SSRibo1 degradation models. Half lives are given for the reaction as a whole where the reaction has multiple steps.
